# Toward a dynamical theory of body movement in musical performance

**DOI:** 10.3389/fpsyg.2014.00477

**Published:** 2014-05-21

**Authors:** Alexander P. Demos, Roger Chaffin, Vivek Kant

**Affiliations:** ^1^Music Performance Lab, Department of Psychology, University of ConnecticutStorrs, CT, USA; ^2^Department of Systems Design Engineering, University of WaterlooWaterloo, ON, Canada

**Keywords:** music performance, dynamical systems, synergy, body movement, musical communication

## Abstract

Musicians sway expressively as they play in ways that seem clearly related to the music, but quantifying the relationship has been difficult. We suggest that a complex systems framework and its accompanying tools for analyzing non-linear dynamical systems can help identify the motor synergies involved. Synergies are temporary assemblies of parts that come together to accomplish specific goals. We assume that the goal of the performer is to convey musical structure and expression to the audience and to other performers. We provide examples of how dynamical systems tools, such as recurrence quantification analysis (RQA), can be used to examine performers' movements and relate them to the musical structure and to the musician's expressive intentions. We show how detrended fluctuation analysis (DFA) can be used to identify synergies and discover how they are affected by the performer's expressive intentions.

## Introduction

Almost universally, musicians sway as they play in ways that appear to be musically expressive. Postural sway reflects emotion (Stins et al., 2011), but understanding of its relationship to musical expression has been hampered by the complexity of the behavior and the lack of suitable tools for studying it. The usual approach has been to adapt the methods and theory used to study language-based gestures (Wanderley et al., 2005; Davidson, 2007, 2012; Ginsborg, 2009). We suggest that this gestural approach has inherent limitations that become more salient when applied to music. We propose a complex systems approach using concepts and mathematical tools developed for describing and analyzing the behavior of non-linear dynamical systems (Kelso, 1995; Latash, 2008).

## Gestural approach to body movement in performance

Linguistic communication is generally thought of as a one-way process in which the listener infers the speaker's meaning from the speech signal (Clark, 1996). The role of gestures is studied by first classifying the different types of body movements that serve as meaningful signals, i.e., gestures, and then seeing how each type of gesture helps convey a speaker's meaning to the listener/perceiver (McNeill, 1992, 2005; Kendon, 1993; Beattie and Shovelton, 1999). Gestures are thought to ground cognition in action (Beilock and Goldin-Meadow, 2010), aid memory retrieval (Cook et al., 2010), provide a window into the speaker's intentions (Goldin-Meadow, 2003), relay emotion (Cavé et al., 1996), and improve intelligibility (Munhall et al., 2004).

Music highlights the limitations of this approach. In language, meaning is largely carried by discrete units (e.g., words and utterances) that can be readily linked to body gestures (e.g., pointing), with which they are closely bound in time (McNeill, 2006). In music, on the other hand, meanings and gestures are less clearly demarcated, and ambiguity and vagueness are more pervasive (Patel, 2008). Turn taking is less salient and communication more continuous. Aspects of communication that seem secondary in language are more salient: the communication of emotion (Juslin, 2005), coordination of activity (Blacking, 1995), strengthening of social ties (Gioia, 2006), and the central role of the body (Davidson, 2007).

## The body in performance

Researchers examining the large-scale body movements that musicians make during performance have looked for one-to-one correspondences between particular types of movement and musical features (e.g., slowing at a cadence; Friberg and Sundberg, 1999). This approach has met with some success for *sound-producing* gestures (i.e., movements that make sound). Skilled performers reliably reproduce minute fluctuations in tempo, dynamics, and timbre by accurately replicating their sound-producing movements across performances (Clarke, 1989). For *sound-accompanying* gestures (i.e., postural sway and other movements that do not directly produce sound), on the other hand, the gestural approach has been less successful, largely because movements seem to differ from one performance to the next (Davidson, 2009). Even so, the conviction that the movements are meaningful persists because they are reliably related to musical structure (Wanderley, 2002; Wanderley et al., 2005; Ginsborg, 2009; Palmer et al., 2009; MacRitchie et al., 2013), convey performers' expressive intentions to audiences (Dahl and Friberg, 2007; Nusseck and Wanderley, 2009), conductors' intentions to orchestras (Luck, 2000), and help musicians coordinate with each other (Goebl and Palmer, 2009; Livingstone et al., 2009; Keller and Appel, 2010; Keller, 2012).

Uncovering the relationship of sound-accompanying movements to musical structure and expression has been hampered by methodological difficulties (Lemann et al., 2009). One problem is that movements in music performance typically serve multiple purposes (Davidson, 2009). A second is that the classification of continuous body movements into discrete types requires arbitrary segmentation that obscures their essential continuity and inter-relatedness. Third, movement is the product of a non-linear system (Latash, 2008). For example, performer's sway is not a simple product of the beat plus expression. Elements interact so that a change in one produces non-linear changes in the other (Davidson, 2002; Wanderley et al., 2005). A complex system approach avoids these problems, providing for one-to-many mappings between actions and goals, avoiding arbitrary segmentation, and respecting the complex inter-relatedness of the motor, cognitive and affective systems.

## Complex systems perspective

Dynamical systems theory provides a systematic approach to the study of complex systems along with the mathematical tools needed to identify regularities in their behavior and track their evolution over time (Strogatz, 1994). These have been successfully applied to action, thought, and social interaction by psychologists working in the cognitive and ecological traditions (Kelso, 1995; van Gelder, 1998; Thelen and Smith, 2006; Warren, 2006; Marsh, 2010; Bruin and Kästner, 2012). Dynamical systems theory has been extensively applied in the field of motor control by treating movement as a continuous, time-evolving process on which multiple constraints are imposed simultaneously by the physical, mental, and social contexts (Latash, 2008).

The behavior of a complex system is a product of its initial conditions, the interaction of its components, and the constraints imposed by the context (Strogatz, 1994). To experience this, point the tips of your two index fingers toward each other. Slowly move your fingers up and down in opposite directions (anti-phase). Slowly speed up to go as fast as you can. As you speed up, you will notice that your fingers spontaneously start moving in the same direction (in-phase; Haken et al., 1985). In contrast, if you start out fast and in-phase and slow down, there is no automatic transition to anti-phase movement. This simple exercise illustrates the self-organizing nature of a complex system. Behavior is an emergent product of initial conditions, components, and constraints (such as movement frequency). Another example is provided by the spontaneous rhythmic entrainment that spontaneously occurs when two people perform a repetitive movement while seated side-by-side (Richardson et al., 2005; Demos et al., 2012). The frequency of their movements is not predictable from their behavior when alone but is an emergent product of their interaction (Miles et al., 2010).

In systems of even modest complexity, components can be organized in an indefinite number of configurations (the degrees of freedom problem; see Turvey, 1990). As a result, there is no one-to-one correspondence between components and functions. The same goal can be accomplished by a variety of different movements; the same movement by a variety of patterns of neural activation (Thelen, 1995). Stability is achieved by temporarily limiting the number of possibilities by constraining parts of the system, allowing the required behavior to emerge from the interaction of muscles, limbs, spine, and brain, and other components both inside and outside the body. These organize themselves, just as your two fingers did, into temporary functional assemblies, called *synergies*, which enable purposeful behavior and recovery from perturbations (Bernstein, 1967; Latash et al., 2007). In social situations requiring joint action, synergies automatically extend across participants (Marsh, 2010; Riley et al., 2011).

A synergy is not simply the linear sum of the activity of its parts, but is a non-summative product of their interaction; it is non-linear (Latash, 2008). Synergies have three main properties: pattern sharing, task-dependence, and trade-offs (Latash, 2008). Pattern sharing refers to the idea that the work required to accomplish a particular goal is distributed across units (e.g., neurons, muscles, people). Task-dependence refers to the idea that a particular functional assembly will be adapted for use in a variety of contexts (e.g., using your hand to turn a knob or a screwdriver). Most important for our purposes is the idea that actions are accomplished by trading-off stability and flexibility. Stability in one part of the system is achieved by increased variability elsewhere. For example, in order to stabilize their position on the two spatial dimensions that must be controlled to hit a target, expert marksmen increase variability on the third, non-essential dimension (Scholz et al., 2000).

## Measurement of dynamical systems

The behavior of a complex system can be difficult to unpack because of its inherent complexity. An early success was Mandelbrot's (1967, 1983) use of fractal mathematics to describe the seemingly random structure of the English coastline. He showed that there is an underlying regularity to the pattern based on *self-similarity* at different scales. The shape of each small region is similar to the larger region in which it is embedded. Self-similarity is a hallmark of complex systems.

We will briefly describe two methods for identifying self-similarity. First, recurrence quantification analysis (RQA) identifies recurrent states, i.e., self-similarities, when the behavior of a system is plotted in phase-space. Phase-space is an abstract mathematical representation of the functioning of a system over time (Abarbanel, 1995, p. 21). The phase-space of any non-linear complex system can be reconstructed from measurement of the system on a single dimension because each dimension contains information about all the other dimensions (Takens, 1981). Recurrence between two systems can be identified in similar fashion using cross-recurrence quantification analysis (CRQA; see Marwan et al., 2007). Second, detrended fluctuation analysis (DFA) quantifies the noise structure in the fluctuations of a time-series (Peng et al., 1994). Complex systems exhibit characteristic noise structures. For example, pink noise indicates the presence of long-range correlations reflecting the presence of processes that operate over time. When extended to different types of time-series and to multiple time-scales this method is called multi-fractal detrended fluctuation analysis (MFDFA; see Ihlen, 2012).

These techniques (RQA, CRQA, and [MF]DFA) have been successfully applied to the analysis of complex systems in many fields (see Marwan, 2008 for RQA; Ihlen, 2012 for MFDFA) and have been recently adopted by psychologists to study change in behavior over time. Typical applications have examined inter-speaker coordination of postural sway (Shockley et al., 2003), eye movements (Richardson and Dale, 2005), and word order (Dale and Spivey, 2005, 2006). For music performance, the techniques have been successfully applied to the timing of actions (Rankin et al., 2009) and postural sway (Demos et al., 2011; Demos, 2013).

## A dynamical approach to gestures in music performance

In order to perform, a musician must interpret the musical structure, organizing the notes provided in the score in terms of phrasing, rhythm, meter, melodic contour, and so on. The musician expresses this understanding through nuances of timing, articulation, dynamics, and timbre (Clarke, 1989, 1995; Kendall and Carterette, 1990; Palmer, 1997). The process creates a complex web of bi-directional (possibly non-linear) relationships between structure, movement, and sound. This is why musicians seem to sway differently each time they play (Davidson, 2009), why dampening musical expression reduces sway (Davidson, 2002) and dampening sway reduces expressive variation in timing (Wanderley et al., 2005). Music performance seems to be the product of a complex system whose components include minimally the score, instrument, performer, and audience (Hargreaves et al., 2005). We will show how techniques designed for dynamical systems reveal additional connections between movement, structure, and expression.

First, we describe the application of MFDFA and RQA to the postural sway of two trombonists as they each played the same two solo pieces twice in each of three different performance styles (normal, expressive, non-expressive), for a total of 24 performances (Demos, 2013). After each performance, the musicians marked the phrasing they had used on a copy of the score. Phrasing changed with the performance style, differently for each performer. For example, when playing expressively one performer used longer phrases, the other shorter. These changes rippled through the system and were reflected in each musician's postural sway.

We measured sway on two spatial dimensions, anterior-posterior (AP) and medio-lateral (ML). Sway in the two directions can be independent (Winter et al., 1996) or coupled (Balasubramaniam et al., 2000; Mochizuki et al., 2006) depending on the requirements of the task. The AP and ML movements of the trombonists were coupled, *R*^2^_(22)_ = 0.41, *p* < 0.001. Also, sway was different in the AP than in the ML direction due to the need to compensate for the back and forth movements of the trombone slide.

**Figure 1:**
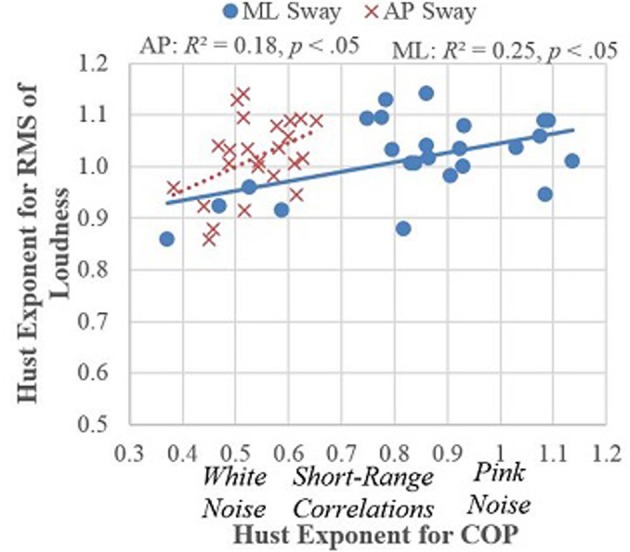
**Hurst exponents for ML and AP sway in 24 performances plotted against RMS of loudness**.

Figure 1 shows how the musical dynamics (fluctuations in loudness) were related to the noise structure of the musicians' movements (obtained by MFDFA). The figure quantifies the relationship between postural sway and musical expression, showing the root-mean-square (RMS) of loudness (a measure of musical dynamics) plotted against the Hurst exponents for the velocity of center-of-pressure measurements of large-scale postural sway, separately for ML and AP directions. Hurst exponents, obtained by MFDFA, measure the quality of the noise in the movements with values close to 1 indicating more long-range self-similarity (pink noise) and smaller values (between 0.5 and 1) indicating self-similarity over shorter ranges, or no correlation (white noise = 0.5). As can be seen, as the sound became pinker (more long-range similarity) the sway moved in the same direction with ML sway becoming pink and AP sway becoming less white. This result quantifies the relationship between postural sway and musical expression that is self-evident to any musician or audience member (Davidson, 2009). While the measures may be unfamiliar, the human senses are attuned to the physical properties they reflect, even though psychological science has been slow to measure them (Van Orden et al., 2003). White noise is the sound of static; pink noise is more structured: the sound of wind in the trees, a musical beat (Rankin et al., 2009), or a melodic pattern (Voss and Clarke, 1978; Su and Wu, 2006).

**Figure 2:**
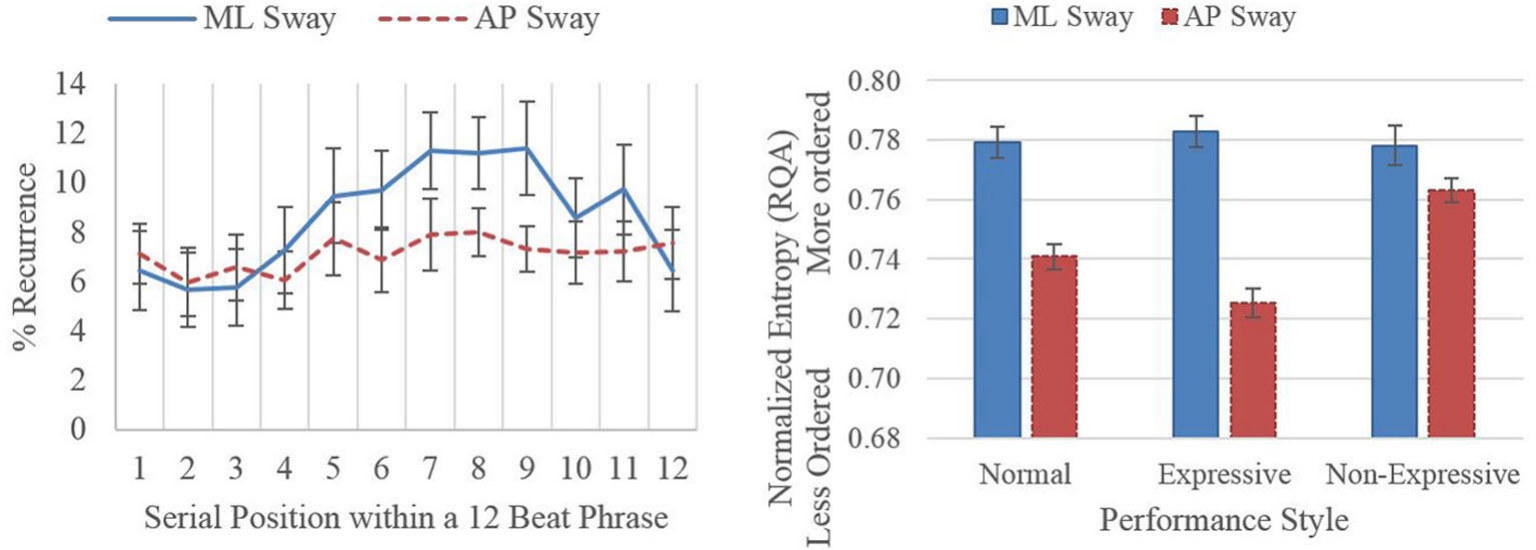
**Left panel displays mean percent recurrence as a function of serial position in a phrase for performances in the normal style**. Right panel displays mean entropy of recurrence for each direction of sway and each performance style.

Figure 2 summarizes two results from the RQA of the performances. Before RQA, we first performed phase-space reconstruction, separately for ML and AP sway, and then used RQA to measure recurrence (self-similarity) and *entropy* (orderliness, predictability, or structure over time; Marwan et al., 2007). We first did the analyses across each entire performance and then, to relate recurrence to the musical structure, we averaged recurrence for each musical beat across performances. The left panel of Figure 2 shows percent recurrence as a function of serial position within a phrase for the four performances played in the normal style. AP sway was not related to position in the musical phrase. ML sway, in contrast, followed a quadratic curve, with less recurrence at the starts and ends of phrases (tested with mixed models). This means that ML sway was more novel (less recurrent or self-similar) at the starts and ends of phrases. Not shown in the figure was the interaction with length of phrase. For longer phrases, the quadric function flipped, becoming more, instead of less, recurrent at the starts and ends of phrases. This suggests how movement might inform an audience of a performer's musical interpretation and expressive intent.

The right panel of Figure 2 shows the mean entropy of movement across the whole performance, separately for each performance style. There was an interaction between direction of sway and performance style. Overall, AP was less orderly than ML sway, reflecting the need for AP sway to compensate for movements of the trombone slide. Together with the coupling of ML and AP sway reported above, the effect suggests a synergistic trade-off between ML and AP sway. Regular swaying in the ML direction may have provided the stability needed to make the rapid adjustments required in AP sway. This interpretation is strengthened by the interaction with performance style. During non-expressive performances, the difference between AP and ML sway decreased due to a sharp increase in entropy for movements in the AP direction. The effect suggests that playing non-expressively changed the synergy, reducing the flexibility of AP movement.

Motor synergies can also be observed in the sounds of musical performance. For example, Chaffin et al. (2007) analyzed the tempo and dynamics of a professional pianist's performances of J.S. Bach's Italian Concerto (Presto). There were more differences between performances at locations important to musical expression (such as structural boundaries), and fewer differences in technically demanding passages. In other words, the pianist exploited the need for a balance between stability and flexibility to achieve both her technical and expressive goals, creating the stability needed to cope with technical difficulties by allowing flexibility at expressively important locations.

The balance between flexibility and stability can be also seen in the sound-producing movements of musicians. When cellists rapidly repeat a note, they reduce variability in the amplitude and duration of movements of the bow and simultaneously increase the variability of movements of the wrist and elbow (Winold et al., 1994). Variability in wrist and elbow buys stability in bowing with speed of bowing acting as the constraint that controls the balance. The balance in bowing can also be affected by the performer's expressive intentions, for example when playing more staccato or legato (Wiesendanger et al., 2006).

The dynamic relationship between musical interpretation, the motor system and expressive interpretation explains why viewers are able to identify the expressive intentions of a performer simply from watching, even when they cannot hear what the performer is playing (Davidson, 1993, 1994, 2007; Nusseck and Wanderley, 2009). It also explains why viewers can identify the emotional intentions of a performer even when they see only head, arm or trunk movements (Dahl and Friberg, 2007). Because the movement of each body part and the musical sounds they produce are all components of the same complex system, each provides information about the others; change in one is related to changes in the others (Latash et al., 2007).

## Conclusion

The study of complex systems is well developed in other fields. Application to the motor system has been amply demonstrated (Kelso, 1995; Latash, 2008). The dynamical systems framework can also help to understand performers' movements and suggests new ways of thinking about the relationship between movement, musical expression, and musical structure.

### Conflict of interest statement

The authors declare that the research was conducted in the absence of any commercial or financial relationships that could be construed as a potential conflict of interest.
